# Cell-Based and Cell-Free Non-Invasive Prenatal Analysis of Preeclampsia: An Updated Review of Liquid Biopsy

**DOI:** 10.3390/biomedicines14040851

**Published:** 2026-04-08

**Authors:** Yafeng Ma, Ya-Wen Chiang, Therese M. Becker, Jon Hyett

**Affiliations:** 1 Obstetrics Research Group, Ingham Institute for Applied Medical Research, 1 Campbell Street, Liverpool, NSW 2170, Australia; 2 South West Sydney Clinical Campuses, School of Clinical Medicine, University of New South Wales, Liverpool, NSW 2170, Australia; 3 School of Medicine, Western Sydney University, South Penrith, NSW 2560, Australia; 4 Centre for CTC Diagnostics & Research, Ingham Institute for Applied Medical Research, 1 Campbell Street, Liverpool, NSW 2170, Australia

**Keywords:** preeclampsia, cell free DNA, circulating trophoblast, fetal nucleated red blood cells, cell free RNA, exosome

## Abstract

Preeclampsia (PE), pregnancy-associated high blood pressure linked to organ damage, affects 3–8% of all pregnancies and results worldwide in 70,000 maternal and 500,000 perinatal deaths each year. Untreated PE may progress to eclampsia with long-term health implications for both mother and child. Non-invasive prenatal diagnosis or screening applies cell-free DNA approaches and offers a less invasive and more economical method for early diagnosis and prediction of various pregnancy complications. Recently, cell-free assays, particularly blood-based cell-free DNA and RNA analysis, have shown great potential in early PE prediction and diagnosis. Here, we provide an updated review of the current understanding and discoveries of PE, focusing on recent publications (1 January 2019–30 December 2025) of liquid biopsy-derived circulating fetal cells (circulating trophoblasts and fetal nucleated red blood cells), cell-free DNA, cell-free RNA and small extracellular vesicles (i.e., exosomes). We aim to discuss the conceptual framework and technical evolution of liquid biopsy applications in preeclampsia pathogenesis, prediction and diagnosis. Progressing novel screening and diagnostic molecular biomarkers have high potential to facilitate early detection for patients at risk of PE. Liquid biopsy-based screening strategies may aid in providing timely intervention and treatment.

## 1. Introduction

**Figure 1:**
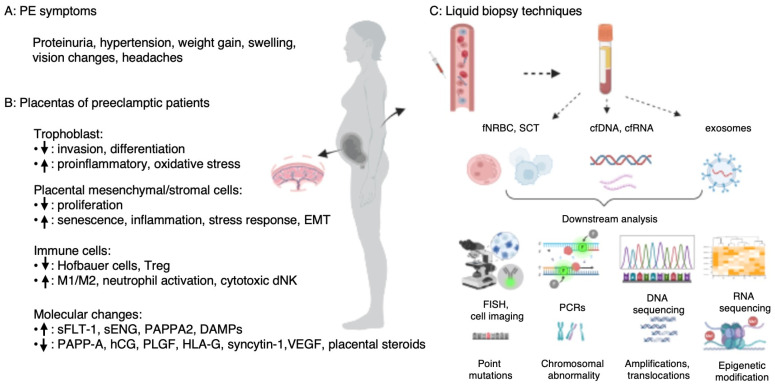
Preeclampsia symptoms, biology and liquid biopsy tools: (**A**) PE symptoms; (**B**) Biology of the preeclamptic placenta. Arrow up means increased and arrow down means decreased; (**C**) Schematic presentation of liquid biopsy tools with genetic analysis applications for prenatal analysis. Note: cf: cell free; DAMPs: damage-associated molecular patterns; dNK: decidual natural killer cell; EMT: epithelial–mesenchymal transition; FISH: fluorescent in situ hybridization; fNRBC: fetal nucleated red blood cell; hCG: human chorionic gonadotropin; HLA-G: human leukocyte antigen G; M1: M1 macrophages; M2: M2 macrophages; PAPP-A: pregnancy-associated plasma protein A; PAPPA2: pappalysin-2; PCR: polymerase chain reaction; PLGF: placental growth factor; sENG: soluble endoglin; sFLT-1: soluble Fms-related receptor tyrosine kinase 1; SCT: single circulating trophoblast; Treg: regulatory T cells; VEGF: vascular endothelial growth factor.

Preeclampsia (PE) is a multi-system hypertensive disorder that occurs during pregnancy, typically after 20 weeks of gestation (GW 20). It is characterized by hypertension (blood pressure ≥ 140/90 mm Hg), proteinuria (>300 mg/24 h urine), and organ dysfunction in the kidney and heart (Figure 1A). PE is one of the leading causes of maternal and perinatal mortality and morbidity worldwide, affecting 3–8% of all pregnancies and resulting in 70,000 maternal and 500,000 perinatal deaths each year [1]. If left untreated, PE can progress to eclampsia, a severe and potentially life-threatening condition characterized by seizures and other complications, with likely long-term health implications for both the mother and the child. Early detection and proactive screening for patients at risk of PE enable timely intervention and management, significantly reducing future healthcare burdens and improving patient outcomes [1]. Liquid biopsy (here generally referring to a maternal blood sample) is emerging as a transformative approach in prenatal diagnosis (e.g., fetal aneuploidy), potentially offering a non-invasive method for early screening for PE and other pregnancy-related complications.

Recent technical advancements in the liquid biopsy and multi-OMICs fields (genomics, epigenomics, transcriptomics, proteomics, etc) have deepened our understanding and insights into placenta development and pathogenesis of PE, thus indicating favorable signs of developing effective strategies for pre-emptive diagnosis and prevention of PE. This review presents a comprehensive update on recent advances (1 January 2019–30 December 2025) in PE-related blood-based non-invasive prenatal analysis. Our focus includes circulating fetal cells, such as fetal nucleated red blood cells (fNRBC) and single circulating trophoblasts (SCTs), as well as cell-free DNA (cfDNA), cell-free RNA (cfRNA), and small extracellular vesicles (sEV or exosomes). We emphasize the challenges and opportunities of applying liquid biopsy approaches to identify novel screening and predictive markers for PE. We anticipate a rapidly growing and dynamic research field centered on cell-based and cell-free analysis related to PE and its potential applications in managing and mitigating other adverse pregnancy outcomes.

## 2. Biology of PE

The underlying mechanisms of PE pathogenesis are mainly unknown. Still, it is thought to be a two-stage process involving stress of fetal syncytiotrophoblast and poor placentation, followed by a second stage of hypoxic placentation, maternal systemic vascular inflammation, and endothelial malfunctions in multiple maternal organs [2]. The categorization is based on the onset timing (early-onset: <GW 34 vs. late-onset: ≥GW 34 or term: ≥GW 37 vs. pre-term: <GW 37 and postpartum: after delivery).

The clinical first-trimester screening tool integrates biochemical (such as serum placental growth factor PLGF) and biophysical factors (such as mean arterial pressure MAP and uterine artery Doppler UAP) with maternal characteristics and medical history. This Fetal Medicine Foundation screening method has been implemented clinically to screen patients at risk in some countries and is effective for predicting early-onset PE (EOPE), with a predictive accuracy of approximately 90%. However, its predictive accuracy for term PE is limited (only 40%) due to the complexity and heterogeneity of the disease [1], underscoring the need for more sensitive and specific screening methods to enhance patient stratification and management. Serum soluble FLT-1(sFLT-1), PLGF and their ratio (sFLT-1/PLGF) are biochemical markers linked to an increased risk of developing PE. Combining such angiogenic or pro-angiogenic biomarkers with first-trimester Doppler sonography (to assess the blood flow in the uterine arteries) may improve the diagnostic performance and predictive value for PE [3]. For patients identified as high risk, administering low-dose aspirin (150 mg/day) before GW 16 can prevent or delay the clinical manifestation of the disease in approximately 80% of patients [4].

Current diagnostic tools for PE primarily rely on the identification of clinical manifestations. In the era of personalized medicine, early diagnosis based on underlying molecular pathology rather than solely on clinical presentations necessitates the identification of novel molecular biomarkers and customizing treatment strategies for specific PE subtypes.

From a biological perspective, single-cell transcriptomics studies of the placenta and decidua [5,6,7,8,9,10,11] revealed the intricate interactions and communications between maternal cells (such as stromal cells and immune cells) and fetal cells (trophoblasts and Hofbauer cells) in the placenta (Figure 1B). For instance, due to a hypoxia environment in a preeclamptic placenta, stromal cells and vasculature present a more inflamed, stress status, while trophoblasts demonstrate impaired invasiveness and differentiation [6,7]. Immune cells shift to a proinflammatory, dysregulated profile with increased cytotoxic natural killer cells, M1 macrophage polarization and reduced regulatory T cells (Tregs) [12]. Additionally, some dysregulated molecules and receptor/ligand imbalances (i.e., sFLT-1 and its ligand PLGF, HLA-G and its respective receptors) play prominent roles in endothelial malfunctions and abnormal maternal immune cell recruitment and tolerance [5,10]. Detailed information on molecular changes and cellular component alterations in the preeclamptic placenta is presented in Figure 1B. Most significantly, such placenta microenvironment and signaling pathways are dynamic and undergo constant evolution throughout gestation, adding further complexity to the understanding of PE pathogenesis and heterogeneity.

PE is often linked to defects in spiral artery remodeling and shallow invasion of the extravillous trophoblasts (EVT) during the first trimester (T1) of decidualization. In patients with PE, the inadequate replacement of maternal endothelial cells with more elastic invasive EVTs (reduced elasticity of spiral artery) causes insufficient blood supply and hypoxia, subsequently triggering cell death signals in trophoblasts and the release of small bodies and vesicles [5,10]. Importantly, trophoblast cell deaths include apoptosis, necrosis, necroptosis [13] and pyroptosis [14], and it is worth mentioning that necroptosis and pyroptosis release large amounts of inflammatory factors, vesicles, DNAs and RNAs to mediate sterile inflammation [14,15]. Also, neutrophils (50–70% white blood cells) undergo inflammatory death pathways through NETosis and releasing neutrophil extracellular traps (network of DNA and proteins), which potentially contributes to the increase in total cfDNA [16]. There is increasing evidence that stress of fetal trophoblasts and maternal chronic inflammation increase the release of cfDNA, cfRNA and extracellular vesicles (EV), providing the foundation for employing liquid biopsy approaches to study PE pathogenesis [17] (Table 1).

Due to ethical considerations, etiological studies of complicated pregnancy and placenta development are predominantly restricted to term or aborted placentas. Also, PE is a pregnancy-associated condition unique to humans and a few primates, and no small animal model has been well validated to replicate PE. These further highlights the critical need to develop liquid biopsy-based methods for understanding PE pathogenesis, screening and diagnosis.

## 3. Liquid Biopsy for Preeclampsia Screening and Diagnosis

Maternal blood-based liquid biopsy offers a minimally invasive alternative to traditional methods such as chorionic villus sampling, amniocentesis, or percutaneous umbilical cord blood sampling. The latter procedures are associated with some risk of miscarriage, infection or bleeding, while liquid biopsy minimizes such risks. It also allows for real-time monitoring of disease progression, comparable to its emerging use in oncology, where longitudinal sampling allows for the detection of biomarker changes related to evolving disease characteristics. Liquid biopsy techniques have demonstrated great clinical utility in prenatal diagnosis [39], cancer diagnosis and prognosis [40], monitoring cancer evolution [40], and assessing transplantation function [41]. The widely used liquid biopsy tools and downstream analysis are illustrated in Figure 1C and listed in Table 1. Although cfDNA-based assay has been widely adopted for the determination of chromosomal aneuploidy, fetal sex and blood type (rhesus status) [42], the diagnostic accuracy (assay sensitivity and specificity) may be suboptimal, false positives exist, and the result is indeterminate when the fetal fraction is <4%.

### 3.1. Cell-Based Approaches

Cell-based assays (analysis of fNRBC and SCTs) may present a promising alternative to allow for non-invasive genetic diagnosis. Fetal cells isolated from maternal blood retain intact fetal genomic information, enabling accurate diagnosis, in some instances, of large deletions and duplications, thus addressing some of the limitations of cfDNA-based methods and improving the overall diagnostic process.

Nucleated RBCs are immature erythrocytes that are typically found in the bone marrow as part of erythropoiesis; they are uncommon in healthy adult circulation but present in fetuses and newborns. Abnormal fNRBC counts in neonate blood are associated with fetal asphyxia and fetal injury [43,44] and PE [45,46]. FNRBC in maternal blood can potentially serve as an ideal candidate for cell-based early screening and diagnosis, as these fetal cells are readily detectable from GW 6, have a limited lifetime (being pregnancy-specific), and are fetus-specific (representing the actual fetal genome rather than the placental genomes as represented by trophoblasts) (Table 1). FNRBC analysis in preclinical research has demonstrated potential in early fetus gender determinations [47], blood ABO genotype [22] and early diagnosis of fetal chromosomal disorders [20]. More male fNRBCs (0.61 ± 1.2 XY cells/mL blood vs. 0.02 ± 0.04 XY cells/mL blood in uncomplicated pregnancies, *p* < 0.001) are found in maternal blood from patients with preeclampsia due to enhanced maternal–fetal cell trafficking [18]. Given that the molecular identity commonly used for adult erythroid cell isolation, such as CD71 and CD235a, is not fetus-specific, further discrimination between fNRBC and potential maternal NRBC may be needed (Table 1). Current research focuses on improving the isolation efficiency, viability, and purity of fNRBC for potential disease diagnosis [21]. An extensive review of fNRBC isolation techniques has been recently published (Table 1) [19]. In summary, the research on fNRBC and total NRBC count in maternal blood and their clinical implications for complicated pregnancies are emerging but need more investigation.

Circulating trophoblasts are circulating EVTs found in maternal blood, detectable as early as GW 6-8 and peak at GW 12 [25]. Various isolation technologies and platforms offer varying success rates and yields to isolate such cells with up to 0.3–1 cell/mL in average maternal blood (Table 1). Trophoblast quantity is inversely correlated with increasing body mass index (BMI) [48]. SCT testing has been validated for detecting fetal aneuploidies and disease-specific genetic markers, showing potential as an early prenatal diagnostic test [26]. Impaired placental function can lead to increased passage of trophoblast cells into maternal peripheral blood (maternal–fetal cell trafficking); thus, the discovery and enumeration of SCT in maternal blood has significant implications in predicting adverse pregnancy outcomes derived from the placenta. Increased SCT number and trophoblast clusters have been discovered in placenta accrete spectrum disorders, which are caused by the deep infiltration of the placenta into the muscle layer of the uterus and abnormal trophoblast invasion into the myometrium [23]. Increased SCT counts in maternal blood are also associated with PE, and HLA-G (a trophoblast marker) positive cells were 6.88 ± 1.54 per 6 mL blood in healthy pregnant patients (*n* = 16) vs. 30.56 ± 5.16 in patients diagnosed with late-onset PE (*n* = 18) [49]. Patients with a higher SCT count have 1.6-fold higher odds of developing hypertensive disorders during pregnancy [24]. The mechanism behind this remains poorly understood, and further research is required to fully understand and define the role that SCT numbers play and their utility in the early detection of PE. In summary, both fNRBC- and SCT-related studies in PE are very limited and not well-validated. Further studies are hindered by the single cell isolation techniques.

### 3.2. Cell-Free Approaches

Cell-free approaches include analysis of cfDNA, cfRNA, and sEV or exosomes. These entities convey genetic, proteomic, and lipidomic information that may be associated with pregnancy disorders. Research based on cfDNA and cfRNA at various stages of gestation reveals distinct components and features of both maternal and fetal origin, mirroring the dynamic progression of placental development.

#### 3.2.1. Cell-Free DNA (cfDNA)

Placenta-derived cfDNA accounts for approximately 5–30% of total maternal peripheral blood cfDNA, depending on gestational age, maternal status, and health condition of mother and fetus [50]. Fetal fraction (FF, prominent peak ~143 bp) is slightly shorter than adult cfDNA (~166 bp), indicating that different DNA cleavage mechanisms are involved [51]. Across multiple PE studies (Table 2), a higher absolute cfDNA concentration [52,53] and a lower FF ratio (placenta-derived: total cfDNA) [54,55] are found to be associated with PE severity and premature delivery. FF ratio is positively correlated with other first-trimester PE predictive markers, PLGF and pregnancy-associated plasma protein A (PAPP-A), but negatively correlated with MAP and the uterine artery pulsatility index [50]. However, it is still unclear whether FF ratio can function as a stand-alone risk factor. Further attempts to utilize the traditional first-trimester screening data to predict PE risk are related to FF ratio and characterizing cfDNA fragment profiles, such as end motifs and nucleosome positioning [56], see Table 2. This well-designed study develops a predictive model by combining blood pressure and BMI with analyzing nucleosome patterns in first-trimester prenatal cfDNA screening data which has been widely used for aneuploidy screening, achieving a performance of area under the curve (AUC) of 0.85 for preterm PE. This study demonstrates a feasible low-cost (routine screening) approach based on the liquid biopsy analysis method [56].

Additionally, mitochondrial DNA (mtDNA) is released under cellular stress and tissue damage. Compared to non-pregnant patients, cellular mtDNA copy number in maternal blood is reduced, while the plasma cf-mtDNA copy number is increased across the three trimesters [57]. The exact explanation is unclear because cellular mtDNA could derive from various cells (living or dead) in whole blood (leucocyte, lymphocytes and platelets), while cf-mtDNA can come from all sources of tissues (blood, placenta, other damaged organs) due to cell death and turn over. Regarding PE, plasma cf-mtDNA concentration is reduced in patients at T3 [58]. This could be partially due to placenta mtDNA changes, while cf-mtDNA could also be derived from NEPtosed neutrophils, especially at late stage of PE when chronic inflammation plays prominent roles. Moreover, there is a lack of consistency in assessing cf-mtDNA copy number or concentration, and total cfDNA concentration is increased in PE situation. Intriguingly, the majority of measurable cf-mtDNA is membrane-bound or vesicle-encapsulated [58], and free-floating cfDNA is degraded quickly, which warrants further study in tissue (e.g., placenta, neutrophil)-specific mtDNA release and clearance, and how tissue specific mtDNA change in complicated pregnancy.

cfDNA size distribution and fragments may also be predictive of PE [30,59,60] (Table 2). A small proportion (8.7% cfDNA) of placental-derived long DNA (>533 bp) was found in maternal plasma, and the amount of long placental-derived cfDNA increased during gestation development. Interestingly, PE patients have a significantly lower percentage of long cfDNA (>500 bp: 6.6% vs. 8.7% in healthy pregnant patients, *p* = 0.014) [61].

Other relevant PE pathogenesis and prediction markers may be the nucleosome footprints and DNA methylation patterns of cfDNA [28,31,62] (Table 2). Expectedly, due to the activity of many developmental genes, placental-derived DNA exhibit a higher degree of hypomethylation and generally different methylation patterns than adult DNA. Specific genes, such as the hypomethylated *SERPINB5* and the hypermethylated *RASSF1A*, can be used as markers for fetal DNA [63]. Given that highly sensitive PCR-based assays can be designed to detect methylation and thus provide an economical method to measure fetal fraction, cfDNA methylation biomarkers are attractive for screening. Hypermethylated *RASSF1A*, which measures placental derived cfDNA, is higher in the PE group (vs. uncomplicated pregnancies) during all studied trimesters and increases significantly in the second half of pregnancy [64]. EOPE-specific hypermethylated sites were found predominantly in the promoter regions and introns, and EOPE-specific methylation haplotypes were enriched in the CTCF motif (target sequence for CCCTC-binding factor, a zinc finger DNA-binding protein) [62]. The depth distribution patterns of promoters (this refers to how promoter regions are distributed across different genomic regions, chromatin states or functional contexts), as determined by low coverage whole genome sequencing on plasma cfDNA from 60 PE patients (vs. 240 controls), can predict PE with an accuracy of 83%, and the study further validated that a set of 10 genes (including NF-*k*B, an inflammation and immune function gatekeeper) perform well as a PE predictor (Table 2) [65]. In summary, such findings of cfDNA studies are limited in research settings to discover new PE biomarkers, further validations are needed before any clinical application.

**Table 2:** A summary of studies on PE pathogenesis, prediction and diagnosis using plasma/serum cfDNA in the last 6 years (2019–2025).

Author, Year	Patients; Sample Type	Study Type (Sampling Time)	Methods	Findings and Implications of PE
Adil et al., 2025 [56]	395 FF-training cohort, 450 PE-training cohort, 831 validation cohort, 141 external validation cohort; plasma and tissues	PE prediction (≤GW 16)	QIAsymphony Circulating DNA Kit and Low coverage (0.5X) WGS for plasma, and ATAC-seq, DNase-seq and ChIP–seq for tissue	A PE prediction model with validated prediction performance (81% sensitivity at 80% specificity) for preterm PE was established based on maternal and fetal tissue signatures (≤GW 16). Lower estimated FF in early PE, while FF increased across gestation in normal pregnancies.
Li et al., 2025 [28]	8 non-pregnant women, 14 healthy, 12 PE pregnancy women; plasma	PE diagnosis	cfDNA WGS	Different nucleosome footprints indicate specific gene expression profiles for different groups. 1978 differential genes predominantly modulate immunology, cell cycle regulation, and sensory perception between healthy and pre-eclamptic pregnancies.
Stanley et al., 2024 [16]	301 healthy controls, 18 PE and 30 healthy; plasma	PE diagnosis	cfDNA deconvolution	Identify major trophoblast (EVT, etc) contributions to cfDNA, establish cell type signature for PE at diagnosis: AFP+ ALB+ cytotrophoblasts and liver neutrophils and monocytes.
Khalil et al., 2024 [55]	72 EOPE, 251 preterm PE, 420 term PE, and 16,849 healthy pregnant women	PE prediction (T1)	An artificial intelligence model, machine learning algorithms for classification	Lower FF and higher total cfDNA in the PE group.
Baetens et al., 2024 [66]	27 PE and 50 healthy women; plasma	PE prediction (GW 11-13), PE diagnosis (GW 24-37), longitudinal study	Maxwell RSC LV ccfDNA kit, bisulfite sequencing	42 distinct early pregnancy DMRs associate with severe PE.
Yu et al., 2024 [67]	143 EOPE, 580 LOPE and 2004 healthy; plasma	PE prediction (GW 12-22)	Machine learning on NIPT data	EOPE women and healthy pregnant controls differed in pTSS coverages of an 8-gene panel. The early and later onset PE classifiers outperformed the FMF predicting model.
He et al., 2023 [62]	135 pregnant and 50 non-pregnant women; plasma and placenta	PE prediction (T1 and early T2)	MagPure cDNA LQ kit, Methylation capture bisulphite sequencing	cfDNA specific methylation haplotypes and nucleosome positioning patterns were established to predict EOPE.
Gekas et al., 2023 [59]	4 EOPE, 8 LOPE, 83 healthy pregnant women; plasma	PE prediction (T1 and early T2)	Illumina’s VeriSeq™ NIPT Solution v2 assay	cfDNA concentration, FF and fragment size distribution are significantly different at T1, while only FF and concentration are different between PE and controls at T2.
Gai et al., 2023 [61]	10 PE, 16 healthy pregnant controls; plasma	PE diagnosis (T3)	QIAamp cNA kit, ddPCR	PE patients have lower median percentage of long cfDNA
De Borre et al., 2023 [60]	498 pregnant women; plasma	PE prediction (T1 and early T2)	Maxwell HT cfDNA kit and methylome profiling	cfDNA methylome predicts PE pre-symptomatically at GW 9-14. Combined risk score predicted 72% patient with EOPE at 80% specificity.
Spinelli et al., 2022 [31]	5 PE and no chromic HT vs. 5 chronic HT vs. 5 controls; serum	PE prediction (T1 and early T2)	MagMAX Cell-Free DNA Isolation Kit and WGBS	significant DMRs and annotated genes imply a common cardiovascular predisposition in PE and HT groups at T1.
Madala et al., 2022 [54]	534 pregnant women; plasma	PE prediction	massive parallel signature sequencing	Low FF is associated with an increased risk of HDP.
Liu et al., 2021 [29]	41 GH, 62 PE, 148 normal pregnancies; plasma	PE diagnosis	qPCR	cfDNA and ST2 concentrations higher in GH and PE patients, cfDNA is not increased in T3.
Kolarova et al., 2021 [52]	20 PE vs. 22 healthy; plasma	PE diagnosis	sequencing	cfDNA fraction did not differ between groups; however, total cfDNA was >10 times higher in PE and associated with early delivery
Karapetian et al., 2021 [64]	20 PE vs. 22 healthy; plasma	PE prediction	PCR based on *RASSF1A* methylation	Higher cfDNA level in the PE group. cfDNA level increased significantly for the three stages during uncomplicated pregnancy, while in the PE group, cfDNA elevation was significant only in the second half of pregnancy
Kwak et al., 2020 [68]	68 HDP vs. 136 controls; plasma	PE prediction (T2), PE diagnosis (T3)	PCR based on methylated *HYP2* genes as total cfDNA marker	Total cfDNA levels as measured with methylated *HYP2* gene can be used to predict EOPE and PE with small for gestational age neonate.
Guo et al., 2020 [65]	2,199 pregnancies (578 with complications vs. 1621 controls); plasma	PE prediction	Low coverage WGS	Classifiers based on nucleosome positioning predict complications with an accuracy of 80.3%, 78.9%, 72.1%, and 83.0% for macrosomia, FGR, GDM, and PE, respectively.
Yuan et al., 2019 [53]	831 pregnant women; plasma	PE prediction (GW 12-22)	KingFisher Flex cfDNA extraction system	Total cfDNA levels were significantly higher in women diagnosed with PE. Increase in cfDNA levels were associated with an increased risk for PE.

Note: AFP: Alpha-fetoprotein; ALB: albumin; AUC: area under the curve; ChIP–seq, chromatin immunoprecipitation and sequencing; ddPCR, droplet digital PCR; DMR: differentially methylated regions; DNase-seq, DNase I hypersensitive sites sequencing; EOPE, early-onset preeclampsia; FF, fetal fraction; FGR, fetal growth restriction; FMF, fetal medicine foundation; GDM: gestational diabetes mellitus; GH, gestational hypertensive; GW, gestational week; HDP: hypertensive disorders of pregnancy; HT, hypertension; LOPE, late-onset preeclampsia; NIPT, non-invasive prenatal testing; pTSS: primary transcription start sites; T, trimester; TAC-seq, Transposase-Accessible Chromatin sequencing; WGBS, whole genome bisulphite sequencing.

#### 3.2.2. Cell-Free RNA (cfRNA)

Similar to cfDNA, cfRNA increases during gestation, with a fetal fraction increasing from <1% at T1 to <4% at GW 18 and about 18% after GW 24 [69]. cfRNA can potentially determine the risk of developing PE well before clinical manifestations. Simultaneous profiling of cell-free messenger RNA (mRNA), miRNA (microRNA) and long non-coding RNA in over 900 samples [70] developed two advanced classifiers for preterm PE and EOPE, demonstrating enhanced performance in terms of positive predictive value and AUC in the validation cohort. With this so far largest patient cohort, the study further demonstrated the downregulation of 8 key miRNAs (MIR130A, MIR144, MIR19B1, MIR215, MIR376C, MIR27A, MIR106A and MIR33A), upregulating 5 PE-relevant target genes (ALB, FGA, LEP, IGFBP5 and SERPINA1). Moufarrej et al. recently identified a panel of 18 genes measurable (either increased or decreased) in cfRNA at T1 that can identify patients at PE risk, and these genes are enriched in the placenta, neuromuscular and immune system [71]. Another cfRNA signature (7 genes: CLDN7, PAPPA2, SNORD14A, PLEKHH1, MAGEA10, TLE6 and FABP1) is reported to predict PE at 14.5 ± 4.5 weeks before delivery with a sensitivity of 75% [35]. Other genes (sFIT-1, SERPINE1, PPBP, IGFBP1, PAPPA, ADAM12, EGF, VEGF, PLAT and OPA1) [34,72] have also been shown to be differentially expressed in cfRNA (see Table 3 for detailed information). Although such findings may be validated in the same study with different cohorts, current findings across studies appear to show no significant consensus on genes, which may be due to differences in extraction and sequencing techniques, as well as ethnic variations, gestation ages in patient cohorts.

miRNA and other non-coding RNA, a group of gene expression and cellular process regulators, are also prominently studied in PE. The C14MC (chromosome 14 miRNA cluster, ch14q32) and C19MC (chromosome 19 miRNA cluster, ch19q13.41) are two placenta-specific miRNA clusters, some individual C14MC and C19MC miRNAs (miR-516, -518, -520 and others) are differentially expressed in PE conditions [74,75,76,77,78], and further miRNA-mRNA regulatory network and placenta-relevant pathways are reported in various studies [79,80,81]. Most studies are single-center investigations, performed with miRNA profiling or real-time PCR on RNAs directly extracted from serum or plasma (Table 4), or isolated exosomes (Table 5). Further miRNA comprehensive review related to PE is available in a recent publication [82]. Key findings of miRNA studies are listed in Table 4. The miRNA studies that explicitly state their exosome resource are listed in Table 5.

#### 3.2.3. Exosome and Exosomal Proteins

Exosomes and other EVs are generally emerging as accessible liquid biopsy components. They mediate communications between donor cells and recipient cells via their unique cargos, such as DNAs, RNAs and proteins. Exosomes are defined as small EVs (<200 nm) of endosomal origin. Although it is recommended to use exosomes only when they can be of biologically defined endosomal origin, researchers who isolate vesicles using various methods (ultracentrifugation, precipitation, size exclusion chromatography) and further characterize cfDNA, miRNA profiling, and proteomics sometimes use both terminologies interchangeably, as vesicles are a mixture of variable-sized particles with a heterogeneous composition, and contaminations always exist. Review of EV synthesis, biological function and size distribution is beyond the scope of this manuscript and can be found in other recent reviews such as [88,89]. Placenta-derived exosomes contribute to maternal immune tolerance by reprogramming circulating maternal monocytes [90]. PE patients, compared to healthy pregnant controls, demonstrate a significant decrease in Treg cell number (3.355 ± 1.546% vs. 4.327 ± 1.597%, *p* = 0.0026) and an increase in Th17 cells (4.129 ± 1.701% vs. 3.276 ± 1.533%, *p* = 0.0098), mediating inflammation and tissue damage in PE [12]. In in vitro models, exosomes isolated from PE patient serum can alter Th17- and Treg-related gene expression and cytokine profiles in peripheral blood mononuclear cells from healthy pregnant patients [12]. Recent reviews of exosome or EV function in normal and PE pregnancy focus on this topic [91,92,93,94]. Exosome or small EV RNA in PE have been widely studied (Table 5). It is worth mentioning that a recent scoping review [94] on EVs in healthy and pathological pregnancies highlights, despite inter-study variations regarding isolation and quantification (lack of standardization), statistically significant exosome number increase in all three trimesters vs. healthy pregnancies, for example, Salomon et al. [95] showed that exosome concentrations in maternal blood from presymptomatic patients who later developed PE increased 2.2-fold relative to the control group over gestation, while healthy pregnant patients have higher exosome quantities than healthy non-pregnant controls.

Exosomes or small EVs may be derived from maternal endothelial cells, leukocytes, platelets, and fetal trophoblasts (placental tissue). Exosomes of endothelial and leukocyte origin are significantly increased in PE, suggesting that vasculature and immune function may contribute to PE development [94,96]. Exosome-derived pregnancy-associated marker proteins placental type alkaline phosphatase, pregnancy zone protein, and other angiogenesis and inflammation-associated proteins have been identified with shotgun label-free proteomics analysis [38]. Given that EVs serve as vehicles for drug delivery and EV cargos are protected from degradation, focusing on EV proteins or nucleic acids (miRNA, etc) may allow the generation of more practical diagnostic and therapeutic assays for PE.

**Table 5:** A summary of studies on exosomes and exosomal cargos in 2019–2025.

Author, Year	Patients; Sample Type	Study Type (Sampling Time)	Methods	Findings and Implications of PE
Than et al., 2024 [76]	24 term PE, 23 preterm PE and 94 healthy controls; plasma	GW 25-31 with 6–7 week before diagnosis	Multiplexed immunoassays for analyzing 82 proteins	While angiogenin, CD40L, endoglin, galectin-1, IL-27, CCL19, and TIMP1 were found to be changed only in the whole plasma fraction, PLGF, PTX3, and VEGFR-1 showed differential abundance in both the plasma and EV fractions in preterm PE.
Gibson et al., 2024 [97]	8 EOPE, 4 LOPE and 14 healthy pregnant controls; plasma	PE prediction (GW 26-32)	miRNeasy Serum/ Plasma Kit, qPCR and Taqman MiRNA RT kit	Exosomal HIF-1α protein and miR-210 were detectable in exosomes. EOPE exosomes carry higher HIF-1a levels vs. controls.
Ghosh et al., 2024 [98]	14 PE and 12 healthy controls; plasma	PE prediction (T1)	miRNeasy Mini Kit, miRNA sequencing	Several C19 and C14 miRNAs were altered in EVs from PE patients. Various miRNAs were identified at T1, T2 and delivery. miRNAs for T1 prediction included miR1307-3p and miR-520a-5p.
Wang et al., 2024 [99]	5 EOPE patients vs. 5 healthy controls, validation: 20 EOPE and 20 healthy controls; plasma	PE diagnosis (GW 30-33)	TrIzol kit and small RNA sequencing, qPCR	miR-7151–5p, miR-1301-3p and miR148b-3p show differential expression.
Ga’l et al., 2024 [81]	6 preterm PE with IUGR and 14 healthy controls, plasma	PE prediction (T1)	exoRNeasy Midi Kit, small RNA seq, and quantitative real-time PCR.	In PE, 16 differentially expressed miRNAs were up-regulated, the six discovered Piwi-associated RNAs had both up- and down-regulated components.
Xu et al., 2024 [77]	Severe PE vs. FGR vs. healthy pregnant women (*n* = 35 each); serum	PE prediction (T1)	miRCURY Exosome Isolation Tissue Kit and qRT-PCR	Placental-derived exosomes exhibited lower levels of miR-520a-5p in both the PE and FGR groups.
Yang et al., 2024 [36]	Severe PE vs. healthy pregnant controls (*n* = 10 each); serum	PE pathophysiology (delivery)	exoEasy Maxi Kit and qRT-PCR	miR-26a-5p, miR-152 and miR-155 were upregulated in the PE vs. control group. miR-18a and miR-221-3p were downregulated (*p* < 0.05).
Navajas et al., 2022 [38]	3 PE vs. 3 healthy pregnant controls; serum	PE pathophysiology (delivery)	qEV Izon exosome isolation, LC-MS/MS	Pregnancy-associated marker proteins (ALPP, PZP) were confirmed from serum exosome.
Li et al., 2020 [37]	20 PE vs. 20 FGR vs. 20 healthy pregnant women; plasma	PE pathophysiology (delivery)	DGU and Taqman miRNA array card	7 exosomal miRNAs were differentially expressed in PE women. Only one exosomal miRNA was also significantly different in whole plasma miRNA analysis.
Pillay et al., 2019 [100]	15 EOPE vs. 15 LOPE vs. 30 healthy controls; plasma	PE diagnosis	Qiagen miRCURY kit, Nanostring ncounter miRNA assay	Higher exosome and placenta associated exosome numbers are related to EOPE and LOPE. Exosomal miRNA signatures associated PE pathophysiology were identified.
Hromadnikova et al., 2019 [78]	102 healthy controls vs. 43 PE vs. 63 FGR vs. 57 GH; plasma	PE prediction (T1)	miRCURY exosome kit and RT-PCR	Selection of C19MC miRNAs with diagnostical potential were tested. The downregulation of miR-517-5p, miR-520a-5p, and miR-525-5p was observed in patients with later occurrence of GH and PE. The predictive accuracy of first trimester C19MC miRNA screening (miR-517-5p, miR-520a-5p, and miR-525-5p) for the diagnosis of GH and PE was significantly higher for expression profiling of maternal plasma exosomes compared to whole plasma.
Dong et al., 2019 [101]	10 non-pregnancies, 20 LOPE, 20 EOPE and 40 healthy pregnancies; plasma	PE diagnosis	miRcute miRNA kit and RT-PCR	Downregulation of miR31 and miR21 is associated with PE.

Note: C19 (or14): chromosome 19 (or 14); C19MC: chromosome 19 miRNA cluster; DGU: density gradient ultracentrifugation; EOPE: early-onset PE; EV: extracellular vesicle; FGR: fetal growth restriction; GH: gestational hypertension; IUGR: Intrauterine growth restriction; LC-MS/MS: liquid chromatography-tandem mass spectrometry; LOPE: later-onset PE; miR: microRNA; qRT-PCR: quantitative real-time reverse transcription PCR.

## 4. Limitations and Future Directions

### 4.1. Technical Challenges in Isolation and Analysis

Non-invasive prenatal testing (NIPT) has seen significant advancements over the last two decades, with widespread adoption in determining common trisomies and sex aneuploidies. However, the applications of maternal blood as a noninvasive tool for prenatal screening or diagnosis remain limited to specific scenarios, as outlined above. Cell-based assays potentially provide detailed genetic information of the fetus, while the isolation requires a laborious process and sophisticated equipment. The platforms initially developed for circulating tumor cell isolation are gaining attention in trophoblast isolation, leveraging the unique biological and physiological properties of circulating trophoblasts (Table 1) [23,27,102]. Meanwhile, fNRBC isolations primarily depend on cell surface antigen expression and gradient separations. The limited quantity of circulating trophoblasts and fNRBCs extractable from a blood sample poses significant challenges for downstream analyses; thus, whole genome (transcriptome) amplification as described for circulating tumor cells [103] and in emerging prenatal research [104] is needed before any downstream sequencing.

Similar technical issues apply to cfDNA, cfRNA and exosome research, where varied isolation and analytical methods and distinct patient cohorts complicate study comparisons. CfRNA (mRNA and miRNA) and cfDNA in plasma or serum are protected from degradation when they are membrane or proteins (such as histones) bound or packaged within vesicles. CfDNA, cfRNA and exosome studies can be conducted on banked plasma or serum samples in batch, whereas trophoblast and fNRBC analyses require fresh blood and specialized machines, which are time- and cost-intensive and further limit the clinical applications of cell-based assays. The biomarkers discovered here are exploratory, and before they can be translated into clinical practice, several factors require standardization in sample collection (serum vs. plasma), storage conditions (temperature and durations), as well as isolation and analysis methods. Overall, liquid biopsy analysis in maternal health is evidently an emerging field, which inevitably means that most studies to date are exploratory (proof of principle nature), or of a pilot study, in mostly small and sometimes heterogeneous cohorts of patients. Therefore, large-scale validation studies with more carefully clinically characterized patients are crucial to establish the utility and reliability of liquid biopsy in predicting adverse pregnancy outcomes.

### 4.2. Innovations in OMIC Technologies and Precision Medicine

Recent advances in epigenomics and methylome of cfDNA [31,60,62], single-cell whole genome sequencing, and cfRNA transcriptomics [70,80], incorporated with placental tissue single-cell RNA sequencing and spatial transcriptomics [6,7], provide profound insights on gestational development and PE pathogenesis. Harnessing multi-omics technologies in the same sample cohort offers the opportunity for a comprehensive interpretation of genetic and molecular landscapes involved in placenta development and maternal–fetal immune tolerance [105]. The distinctive advantage of liquid biopsy lies in its ability to longitudinally monitor patients across different trimesters and postpartum stages, which is impractical using more invasive biopsies. Thus, liquid biopsies promise significant and unique insights into obstetrics research. Importantly, the application of cfDNA methylome and cfRNA transcriptome classifiers, either alone or in combination with other prenatal screening or predictive models, has demonstrated improved diagnostic accuracy and reliability in multiple studies preclinically [60,70].

Current clinical approaches for PE prediction and diagnosis primarily rely on maternal risk factors, close monitoring and assessment of blood pressure, proteinuria and angiogenesis markers (sFLT-1/PLGF ratio). These methods are widely implemented for short-term risk stratification in symptomatic patients; however, their predictive performance in early gestation remains moderate and largely reflects downstream placental dysfunction rather than early molecular alterations. The Fetal Medicine Foundation algorithm clinically predicts PE risk at early stage; while it is developed and validated primarily in European cohorts, the performance may vary in other low- and middle-income settings. The algorithm performance highly relies on the accuracy of other measurements (PLGF, PAPP-A, MAP and Doppler). There is also time restriction (GW 11-13, single time point assessment) and resource constraints associated with the algorithm clinical applications. In contrast, analysis of cfDNA, cfRNA, and exosome-derived cargo aims to dynamically capture placental and maternal molecular signatures preceding clinical manifestation. Emerging studies suggest that quantitative and qualitative changes in cfDNA (e.g., concentration, fragmentomics, methylation patterns), placenta-specific cfRNA transcripts, and exosomal miRNAs may enable earlier risk prediction and improved mechanistic insight, with an additional, potentially superior predictive value. Nevertheless, these advanced platforms remain heterogeneous in assay design, normalization strategies, and cohort validation, and their diagnostic performance varies across studies. Therefore, while conventional angiogenic markers remain the most clinically actionable tools at present, liquid biopsy technologies represent a promising next-generation strategy with potential for earlier detection and improved biological stratification, pending further standardization and large-scale validation. Thus, incorporating liquid biopsy with other non-invasive maternal tests, such as sonography and biochemical biomarkers, will eventually facilitate precision medicine.

### 4.3. Limitations Across Various Studies and Future Prospectives

The reviewed studies underscore the promise of liquid biopsies for elucidating PE pathogenesis and for non-invasive screening. However, several limitations must be acknowledged. A key constraint is that most evidence derives from case-control studies, which limits the generalizability of the findings. For clinical translation, future research must prospectively validate novel cfDNA/cfRNA markers and benchmark them against established screening tools to confirm their incremental value. Furthermore, classifying PE by placental pathology [106], or with or without fetal growth restriction (FGR), in addition to clinical presentation (onset timing and disease severity), might improve patient stratification. Finally, the broad implementation of NIPT offers a pivotal opportunity; leveraging this data with machine learning and artificial intelligence models [107,108] could potentially enable cost-effective screening for PE, while further studies on other placental complications like FGR are warranted.

The release of fetal cells and cell-free components is not unique to PE; similar phenomena are observed in other pregnancy-related complications. CfDNA and cfRNA are also implicated in FGR, although comparatively fewer studies have investigated this association. In gestation diabetes mellitus, cell free entities, especially miRNA and cfDNA, are extensively studied, with miRNAs showing considerable promise as biomarkers for early diagnosis [109,110,111,112]. Future research should focus on systematically comparing cell-free entities across different pregnancy complications to determine whether disease-specific molecular signatures exist and to evaluate their potential for early diagnosis, risk stratification, and mechanistic insight into placental dysfunction.

## 5. Conclusions

Although liquid biopsy in PE is still in its initial stages, recent research findings in cell-based and cell-free approaches are encouraging, illuminating the potential of applying liquid biopsy as a screening and diagnostic tool. Larger-scale clinical trials are required to enable clinical incorporation. Future technical innovations in liquid biopsy and OMICs will pave the way for a better understanding of placenta development, PE pathogenesis, and precision medicine for adverse pregnancy outcomes. Once reliable biomarkers have been identified and validated at a large scale, it is important to develop practical screening or diagnostic tests that can easily be performed in most pathology laboratories to progress clinical translation.

## Figures and Tables

**Table 1:** Summary of cell-based and cell-free approaches.

	Enrichment/ Isolation Methods	Markers or Features	Limitations and Advantages	Downstream Analysis
Cell based				
fNRBC [18,19,20,21,22]	Density gradient centrifugation; silica microbeads; FACS and MACS; immunoaffinity microfluidic chips, nanomaterials	Several to tens per mL blood; early detectable; erythroblast markers include CD71, CD147, glycophorin A, ε-HbF and gamma-HbF	Fragile and short-lived cells; long procedure; markers are erythroblast specific but not fetal specific; morphologically distinct from maternal cells	Enumeration, FISH, dPCR and RT-PCR, array CGH, WGA and WGS
SCT [23,24,25,26,27]	MACS plus FACS; size-based filtration (Metacell); immunoaffinity microfluidic (NanoVelcro chips); single cell picker	One to a few per mL; early detectable; fetal specific markers include HLA-G, hCG, endoglin, cytokeratins	Rare; long procedure; potential placental mosaicism	Enumeration, FISH, dPCR and RT-PCR, CGH, WGA and WGS
Cell free				
Cell free DNA [28,29,30,31]	Commercially available kits from Qiagen QIAamp, Norgen Biotek, Promega Maxwell RSC, Macherey-Nagel	Early detectable, fragment size and epigenetic features vary	Confined placental mosaicism; low predictive accuracy is associated with low fetal fraction; relatively simpler procedure	Fragment analyser, PCR, STR analysis, whole genome bisulphite sequencing, target sequencing
Cell free RNA [32,33,34,35]	Commercially available kits, generally same as cfDNA	Early detectable, multi-types (mRNA, miRNA, lncRNA)	Tend to degrade, potential contamination from platelets, relatively simpler procedure	RT-PCR, Nanostring nCounter for miRNA profiling, transcriptomic profiling, PALM-seq
Exosome [36,37,38]	UC, SEC, PEG precipitation, membrane-filtration, Immunoaffinity capture	Variable size; exosome specific marker CD63 and others, placenta derived exosome marker PLAP and HLA-G	Potential contamination from other particles	RT-PCR, miRNA profiling, EM, WB, NTA, etc

Note: CGH: comparative genomic hybridization; EM: electron microscope; FACS: fluorescence-activated cell sorting; FISH: fluorescence in situ; lncRNA: long non-coding RNA; MACS: magnetic-activated cell sorting; mRNA: messenger RNA; miRNA: microRNA; NTA: nanoparticle tracking analysis; PALM-seq: polyadenylation ligation-mediated sequencing; dPCR: digital polymerase chain reaction; PEG: polyethylene glycol; PLAP: placental alkaline phosphatase; RT-PCR: real time quantitative PCR; SEC: size-exclusion chromatography; STR: short tandem repeat; UC: ultracentrifugation; WB: Western blotting; WGA: whole genome amplification; WGS: whole genome sequencing.

**Table 3:** A summary of studies on plasma/serum cell free RNA in the last 6 years (2019–2025).

Author, Year	Patients; Sample Type	Study Type (Sampling Time)	Methods	Findings and Implications of PE
Gong et al., 2025 [33]	39 PE with FGR, 156 controls	PE prediction, longitudinal study	QIAamp^®^ Circulating Nucleic Acid Isolation, whole transcriptome sequencing, machine learning	Leptin and pappalysin 2 cfRNA are the strongest predictors with AUC of 0.82 each and an AUC~0.951 of combined performance in validation cohort.
Castillo-Marco et al., 2025 [32]	42 EOPE, 43 LOPE and 131 controls	PE prediction (T1), longitudinal study sampling at T1, T2 and diagnosis	MiRNeasy Serum/Plasma Advanced Kit, cfRNA sequencing	A predictive model for EOPE (at T1) consisting of 36 cfRNA transcripts achieved sensitivity of 83% and specificity of 90% with an AUC of 0.88, while the predictive model for LOPE shows limited performance. For PE prediction at T2, the models to predict EOPE with 87 cfRNA transcripts and LOPE with 92 cfRNA are established and further validated.
Pei et al., 2025 [34]	11 EOPE, 53 LOPE and 105 healthy pregnant control; plasma and placental tissues	PE prediction (T1)	CfRNA isolation, qPCR and Transcriptome analysis	Serum and placental tissues from PE patients at different gestational weeks show a substantial increase in transcripts of mitochondrial dynamin-like GTPase (OPA1). Combination of OPA1 levels and MAP yielded an AUC of 0.825 (95% CI: 0.759–0.879) for predicting PE.
Chen et al., 2024 [72]	Cohort 1: 31 PE and 20 controls, cohort 2: 11 PE and 17 controls; plasma	PE prediction and diagnosis	QIAamp^®^ Circulating Nucleic Acid Isolation Kit, qPCR	Establish a nine gene panel. The model combined cfRNA and ultrasonographic indicators to achieve high AUC of 0.91 and sensitivity of 1.0 at T1.
Zhou et al., 2023 [70]	715 healthy and 202 PE patients; plasma	PE prediction (GW 12-33)	Trizol cfRNA extraction and PALM-seq	DEGs are generally mRNA and miRNA, associated with known PE etiology. 2 classifiers and 2 clinical features show strong performance in predicting preterm and EOPE. Down regulation of miRNAs up-regulate PE relevant target genes. Biggest patient cohort to-date.
Seydabadi et al., 2023 [73]	PE vs. normal (*n* = 20 each); plasma	PE prediction (GW 14, GW 28)	QIAamp cfDNA kit and RT-PCR	Significant higher expression of TIMP1-4 in the PE women (vs. controls)
Moufarrej et al., 2022 [71]	Discovery: 49 normotensives, 24 with PE; Validation1: 32 normotensive, 7 PE; Validation2: 61 normotensives, 26 PE	PE prediction and pathogenesis (≤ GW 12, GW 13-20, ≥GW 23, post-partum), longitudinal	Norgen plasma/serum circulating and exosomal RNA purification kit, SMARTer Stranded Total RNAseq kit V2	A reduced placental signal occurs in early gestation of PE, and platelets and endothelial cells drives cfRNA changes before GW 20, immune system demonstrate marked shift changes. A panel of 18 genes identify patients at risk of PE at T1.
Rasmussen et al., 2022 [35]	1840 pregnancies and 2539 banked samples; plasma	PE prediction (GW 14.5 ± 4.5 before delivery)	Norgen RNA kit and cfRNA sequencing	cfRNA robustly predicts PE, with a sensitivity of 75% and a PPV of 32.3%.

Note: AUC: area under the curve; EOPE: early-onset PE; FGR: fetal growth restriction; GW, gestational week; LOPE: late-onset PE; PPV: positive predictive value; T, trimester; TIMP: tissue inhibitors of metalloproteinases.

**Table 4:** A summary of studies on miRNAs in the last 6 years (2019–2025).

Author, Year	Patients; Sample Type	Study Type (Sampling Time)	Methods	Findings and Implications of PE
Senousy et al., 2024 [83]	82 PE vs. 78 healthy pregnant women; serum	PE diagnosis	Qiagen miRNeasy Serum/Plasma kit and RT-qPCR	Lower H19 levels and higher miR-29b levels when EOPE vs. LOPE or control vs. PE. H19 (AUC = 0.818, 95%CI = 0.744–0.894) and miR-29b (AUC = 0.82, 95%CI = 0.755–0.885) are potential EOPE diagnostic markers.
Ping et al., 2023 [80]	PE vs. healthy (*n* = 3 each); plasma	PE diagnosis	TRIzol reagent and mRNA whole transcriptome sequencing and small RNA sequencing	miRNA-mRNA regulatory network was revealed. 51 significantly upregulated miRNA and 19 significant downregulated miRNAs were identified in PE.
Morey et al., 2023 [84]	123 pregnant women; serum	GW 20-40 with suspected PE for PE diagnosis	Norgen Biotek kit and Small RNA-seq	Three bivariate miRNA biomarkers (miR-522-3p/miR-4732-5p, miR-516a-5p/miR-144-3p, and miR-27b-3p/let-7b-5p), when applied serially, distinguished between PE cases of different severity and from controls with a sensitivity of 93%, specificity of 79%, PPV of 55%, and NPV of 89%.
Mirzakhani et al., 2023 [85]	110 healthy controls vs. 47 PE; whole blood	PE prediction (GW 10-18)	Norgen Biotek kit and OpenArray miRNA profiling with RT-PCR	16 differentially expressed miRNAs and 32 unique targets of miRNA signatures were identified.
Lip et al., 2020 [79]	Nonpregnant vs. pregnant vs. EOPE (*n* = 10 each); plasma	PE diagnosis	miRNA 3.1. arrays (Affymetrix) and RT-PCR	Top 3 differentially expressed miRNAs are miR-574-5p, miR-1972, and miR-4793-3p, which regulate endothelial cell functions (proliferation and tube formation).
Jelena et al., 2020 [74]	17 healthy vs. 19 PE patients; plasma	PE pathophysiology (GW 20-39)	QIAzol RNA extraction and DDPCR	miR-518b was significantly elevated in PE (vs. healthy controls).
Demirer et al., 2020 [86]	48 EOPE vs. 48 LOPE vs. 52 healthy controls; periphery leucocytes	PE diagnosis	RT-PCR	miR-518b was upregulated in the EOPE and LOPE groups, compared to controls.
Youssef et al., 2019 [87]	30 PE vs. 20 healthy controls; serum	PE diagnosis	RT-PCR	Higher levels of miR-210 and miR-155 in the PE vs. control group.
Sekar et al., 2019 [75]	50 PE vs. 50 healthy pregnant women; blood	PE diagnosis	RT-PCR	miR-510 is upregulated in PE blood samples and is correlated with promoter methylation.

Note: AUC: area under the curve; CI: confidence interval: EOPE: early-onset PE; GW: gestational week; LOPE: later-onset PE; miR, miRNAs; NPV: negative predictive value; PPV: positive predictive value.

## Data Availability

No new data were created or analyzed in this study. Data sharing is not applicable to this article.
